# Exosomes for gene therapy effectively inhibit the endothelial-mesenchymal transition in mouse aortic endothelial cells

**DOI:** 10.1186/s12891-021-04896-0

**Published:** 2021-11-30

**Authors:** Zhenyuan Wei, Yang Zhao, Peichun Hsu, Shang Guo, Chi Zhang, Biao Zhong

**Affiliations:** Department of Orthopedic Surgery, and Shanghai Institute of Microsurgery on Extremities, Shanghai Jiao Tong University Affiliated Sixth People’s Hospital, Shanghai, 200233 China

**Keywords:** Gene therapy, SMAD7, exosome, endothelial-mesenchymal transition, mouse aortic endothelial cells, endothelial cell targeting property

## Abstract

**Background:**

Heterotopic ossification (HO) can limit joint activity, causes ankylosis and impairs the function and rehabilitation of patients. Endothelial to mesenchymal transition (EndMT) plays an important role in the pathogenesis of HO, and high expression of SMAD7(Mothers Against Decapentaplegic Homolog 7) in endothelial cells can effectively reverse the TGF-β1 mediated EndMT. This article studied an appropriately engineered exosome with high biocompatibility and good targeting property to administrate SMAD7 gene therapy to inhibit the EndMT.

**Methods:**

Exosomes from mouse aortic endothelial cells were cultured and harvested. DSPE-PEG and antibody CD34 were combined to exosomes to synthesize the endothelial cell targeting exosome vector (Exosome-DSPE-PEG-AbCD34). The biocompatibility, stability, targeting and cell internalization of exosome vector were tested, then the Exosome-DSPE-PEG-AbCD34 was loaded with *Smad7* plasmid and administrated to MAECs to examine its therapeutic effect on EndMT of MAEC mediated by TGF-β1.

**Results:**

The Exosome-DSPE-PEG-AbCD34 has no impact on MAEC cell viability at high concentration, and exosome-DSPE-PEG-Ab_CD34_ could be stably stored at 4°C and 37°C for at least 8 days. Exosome-DSPE-PEG-Ab_CD34_ has better targeting property to MAEC cells and can enter into the cells more effectively. The Exosome-DSPE-PEG-Ab_CD34_*-Smad7* could significantly increase the level of SMAD7, decrease the expression of TGF-β1, and effectively reverse the EndMT of MAEC mediated by TGF- β1 in MAEC cells.

**Conclusions:**

The synthesized Exosome-DSPE-PEG-AbCD34*-Smad7* has good biological properties and can effectively reverse the EndMT of MAEC mediated by TGF-β1. Thus, Exosome-DSPE-PEG-AbCD34*-Smad7* may has the potential for the prevention and treatment of HO.

**Supplementary Information:**

The online version contains supplementary material available at 10.1186/s12891-021-04896-0.

## Background

Heterotopic ossification (HO) is an abnormal process of bone formation that occurs in muscle or connective tissue [1]. HO can lead to pain, decrease in the range of motion of adjacent joints, peripheral nerve compression, and pressure ulcers. HO also has a high morbidity rate, ranging from 10% to 36%. These factors make it become one of complications affecting the functional rehabilitation of elbow and hip fracture patients. Therefore, identifying methods to prevent and reverse the pathological process of HO has great economic and social value [2]. Previous studies have shown that progenitor cells in local soft tissue affected by inflammation niches and signaling pathways gradually differentiate into chondrocyte phenotype and osteoblast phenotype and finally form mature bone tissue to produce ectopic bone [3]. However, the pathological mechanisms and related signaling pathways of HO are still unclear [4, 5], and its prevention and treatment methods are limited.

Recent studies have shown that the endothelial mesenchymal transition (EndMT) pathway is important in the pathogenesis of HO [6, 7]. Endothelial markers were reported to be expressed in chondrocytes and osteocytes in ectopic bone of an HO animal model, which were different from normal osteocytes and chondrocytes, indicating that endothelial cells are associated with the occurrence and development of HO [8, 9]. Lounev et al. also proved that the stimulation of inflammatory mediators and signals in local tissue induce vascular endothelial cells transform into chondrocytes and osteocytes through the EndMT pathway, and form ectopic ossification via intramembrane osteogenesis and chondrogenic osteogenesis [10]. Therefore, EndMT can be used as a target to develop prevention and treatment methods for HO. There are a plenty of studies have found that the transcription products of *Smad7* gene can competitively bind to type II TGF-β receptor, thus inhibiting the TGF-β signaling pathway and playing a negative regulatory role [11]. Our previous finding also indicated that SMAD7 overexpression in vascular endothelial cells can effectively block the TGF-β mediated EndMT signaling pathway and thereby attenuate HO. However, SMAD7 overexpression in vascular endothelial cells requires gene vectors with high biocompatibility, good sustained-release performance, high plasmid loading and good targeting properties. Lentiviral vector is a widely used gene vector, but it is difficult to use in clinical treatment due to the limitations of its targeting properties and biosafety.

Exosomes , a sort of saccular vesicles with a diameter of 30-150 nm secreted by living cells, are important mediators of "material exchange" as well as information transmission between cells [12, 13]. As a natural endogenous substance transporter, exosomes have the advantages of low toxicity, no immunogenicity and good permeability and are thus superior carriers for effective molecule delivery [14, 15]. At present, exosomes have been successfully loaded with small molecule such as drugs, miRNAs, genes and proteins for the treatment of cancer, Alzheimer's disease and other diseases [16–21]. Endothelial cells are now one of the most popular cell sources of exosomes due to their high purity and productivity, ease of availability, and lack of ethical issues. Exosomes produced by the endothelial cells share the similar marker molecules with progenitor cells and contribute to the physiological functions of endothelium as well as the pathogenesis of cardiovascular disease. Thus we selected exosomes derived from mouse aortic endothelial cells as the vehicles of gene therapy [12, 22].

In this study, we bound the DSPE-PEG-AbCD34 (antibody of CD34) to the surface of secretory exosomes to synthesize exosome gene vector (Exosome-DSPE-PEG-AbCD34) with high endothelial cell targeting property. Then we loaded the *Smad7* plasmid into the Exo-DSPE-PEG-AbCD34 and examined its effect on the TGF-β mediated EndMT pathway. Our study thus provides a new approach for the clinical prevention and treatment of HO.

## Materials and Methods

### Cell culture

Mouse aortic endothelial cells (MAEC) were purchased from ATCC. The cells were cultured in DMEM medium containing 10% FBS and 5% CO_2_ at 37°C. When the cell density reached 85%, the cells were digested with trypsin solution and passaged.

### Exosome isolation

The exosomes of MAEC were extracted using the Invitrogen™ Total Exosome Isolation kit according to the manufacturer’s instructions. Briefly, 1ml of cell-free culture media was mixed with 500 μl Exosome Isolation reagent and incubated at 4°C for overnight. The mixed solution was centrifuged at 10,000 × g for 1 hour, supernatant was discarded and exosome pellets were resuspended for the following experiments.

### Preparation of Exo-DSPE-PEG-Ab_CD34_

As exosome membrane, mainly composed of lipid membrane, is rich in cholesterol, sphingomyelin and ganglioside, liposoluble compounds are therefore easy to be integrated into exosome membrane [23]. DSPE-PEG-Ab_CD34_ was synthesized by conjugating CD34 antibody to DSPE-PEG2000-NHS. Then the exosomes were treated with DSPE-PEG-Ab_CD34_. The lipophilic DSPE was fused into the exosome membrane, meanwhile the PEG-Ab_CD34_ was carried to the exosome surface. The procedures were as follows: firstly, DSPE-PEG-Ab_CD34_ was synthesized by the reaction of DSPE-PEG2000-NHS with excessive Ab_CD34_ at room temperature for 24 hours. Then the prepared exosomes were dispersed in PBS, and the exosome protein content was adjusted to 50 mg/ml. 0.1 mL DSPE-PEG-Ab_CD34_ solution with a concentration of 20 mg/mL was added to 2 ml of exosome dispersion, then the mixture was gently blown and incubated at room temperature for 1 hour. After that, the dispersion solution was transferred into a special centrifuge tube and ultracentrifuged at 120,000g and 4 °C for 70 min. The upper solution was sucked away and the unbound DSPE-PEG-Ab_CD34_ was removed. The precipitate obtained was Exo-DSPE-PEG-Ab_CD34_ connected with Ab_CD34_. The precooled PBS solution was added to the precipitate to blow and dissolve again for standby.

### Transmission electron microscope (TEM) detection

The purity and structural integrity of exosomes were determined by transmission electron microscopy (TEM), With the following procedure: The protein content of exosome dispersion was diluted to 100 mg/ml with HEPES buffer, then 10 uL exosome dispersion was added to 200 mesh copper mesh (covered with carbon film) for transmission electron microscopy, and the copper mesh was placed in a desiccator at room temperature until completely dried. Then samples were observed under the accelerated voltage of 75 kV, and photos were taken at the same time.

### Detection of exosome particle size

First, the protein content of exosome dispersion was adjusted to 50 mg/mL, and 20 uL of exosome dispersion was added to 980 uL high ddH2O, then the particle size of exosome was measured by a potential particle size analyzer, based on a light scattering method.

### CCK-8 assay

The concentration of logarithmic phase cells was adjusted to 5×10^4^ cells/ml. 100 uL cell suspension was added into each well of a 96 well culture plate, and incubated in 5% CO_2_ incubator at 37°C for 24 h. After drug treatment, the supernatant was discarded, and 100 uL of fresh medium was added to each well, followed by 10 uL of CCK-8 solution. After 4 hours of culture at 37°C, the absorbance (OD) value of each well at 450 nm was detected by an enzyme reader.

The cell viability rate was calculated by the formula: Cell viability % = (OD sample - OD blank) / (OD control - OD blank) × 100.

### Laser confocal microscopy detection

The cells were seeded in 96 well plates at the initial density of 1 × l0^5^ cells per well. The cells were incubated with Exosome-DSPE-PEG-Ab_CD34_ at 37°C for 2 h, then cultured in 60 nm lysotracker red medium at 37°C for 30 min, washed three times with cold PBS, and fixed with 4% paraformaldehyde for 20 min. Then, cells were treated with DAPI for 10 min and washed twice with PBS. The cells were observed by confocal laser scanning microscope (Olympus FluoView fv-1000, Olympus optics Co., Ltd., Tokyo, Japan) and observed with imaging software. The number of FITC positive cells in 10 visual fields in each group was counted, and the mean fluorescence of FITC intensity was calculated by use of imageJ 2.0.

### qPCR assay

The total RNA was extracted using the Trizol (Invitrogen) kit according to the manufacturer’s instructions, then the mRNA was reversely transcribed into cDNA according to the manufacturer’s instructions of the TaqMan microRNA reverse transcription kit. The expression of each gene was detected using Applied Biosystems 7500 fluorescence quantitative PCR. The primers used were provided by SANGON Biotech (Shanghai) Co., Ltd. The primers designed in this paper are shown in Table 1.

**Table 1:** The qPCR primers used in manuscript

Genes	Forward Primer (5' to 3')	Reverse Primer (5' to 3')
*Smad7* *CD31*	5'-CCCGGCGGCGAGGACGAGGAG-3' 5'-GGAGGTGACAGAAGGTGGGATTG-3'	5'-GGATGGTGGTGACCTTTGGCAC-3' 5'-GCTTGGCAGCGAAACACTAACAGG-3'
*VE-cadherin*	5'-CTTCACCCAGACCAAGTACACA-3'	5'-AATGGTGAAAGCGTCCTGGT-3'
*N-cadherin*	5'-GTGCCATTAGCCAAGGGAATTCAGC-3'	5'-GCGTTCCTGTTCCACTCATAGGAGG-3'
*Vimentin*	5'-GGACCAGCTAACCAACGACA-3'	5'-AAGGTCAAGACGTGCCAGAG-3'
*Gapdh*	5'-AGTGCCAGCCTCGTCTCATA-3'	5'-GAGAAGGCAGCCCTGGTAAC-3'

### SDS-PAGE gel electrophoresis and Western Blot

SDS-PAGE gel electrophoresis was used to detect Ab_CD34_ on Exosome-DSPE-PEG-Ab_CD34_, then an appropriate amount of RIPA cell lysate was added to the precipitate of exosome after 12,000g centrifugation and then incubated in an ice bath for 25 min. It was then centrifuged at 10,000g for 20 min. The BCA protein quantitative kit was used to quantify the protein concentration of exosomes and cell lysates. The lysate containing the same amount of protein was mixed with the SDS-PAGE sample loading buffer, heated to 100°C and kept for 5 min. The protein in the above mixture was separated by SDS-PAGE electrophoresis, then stained with R250 gel dyeing solution, and then decolorized with the decolorizing solution. The exosome marker proteins (CD9, CD63) [24], SMAD7, CD31, VE-cadherin, N-cadherin, vimentin and GAPDH were detected by western blot assay. Exosomes and cells were lysed by RIPA lysate, and after 12,000g centrifugation, the protein of exosomes and cell lysate was quantified using the BCA protein quantitative kit. The lysate containing the same amount of protein was mixed with the loading buffer to prepare samples. The protein samples were separated by SDS-PAGE and analyzed by western blot, then the protein was transferred to PVDF membrane by blot, and then sealed with 5% skim milk for 1 hour at room temperature. The first antibody corresponding to the detected protein was added and incubated at 4°C for 4 hours. After reacting with HRP labeled secondary antibody and washing, ECL luminous solution was used for exposure and photo recording.

### Data analysis and statistical methods

Statistical data were expressed as mean ± SD, and one-way analysis of variance (ANOVA) was used for statistical analysis. *P < 0.05, * * *P* < 0.01 and * * *P* < 0.001 were regarded as significant differences. SPSS 18.0 software was used for statistical analysis.

## Results

### Preparation and characterization of Exosome-DSPE-PEG-AbCD34

**Fig. 1:**
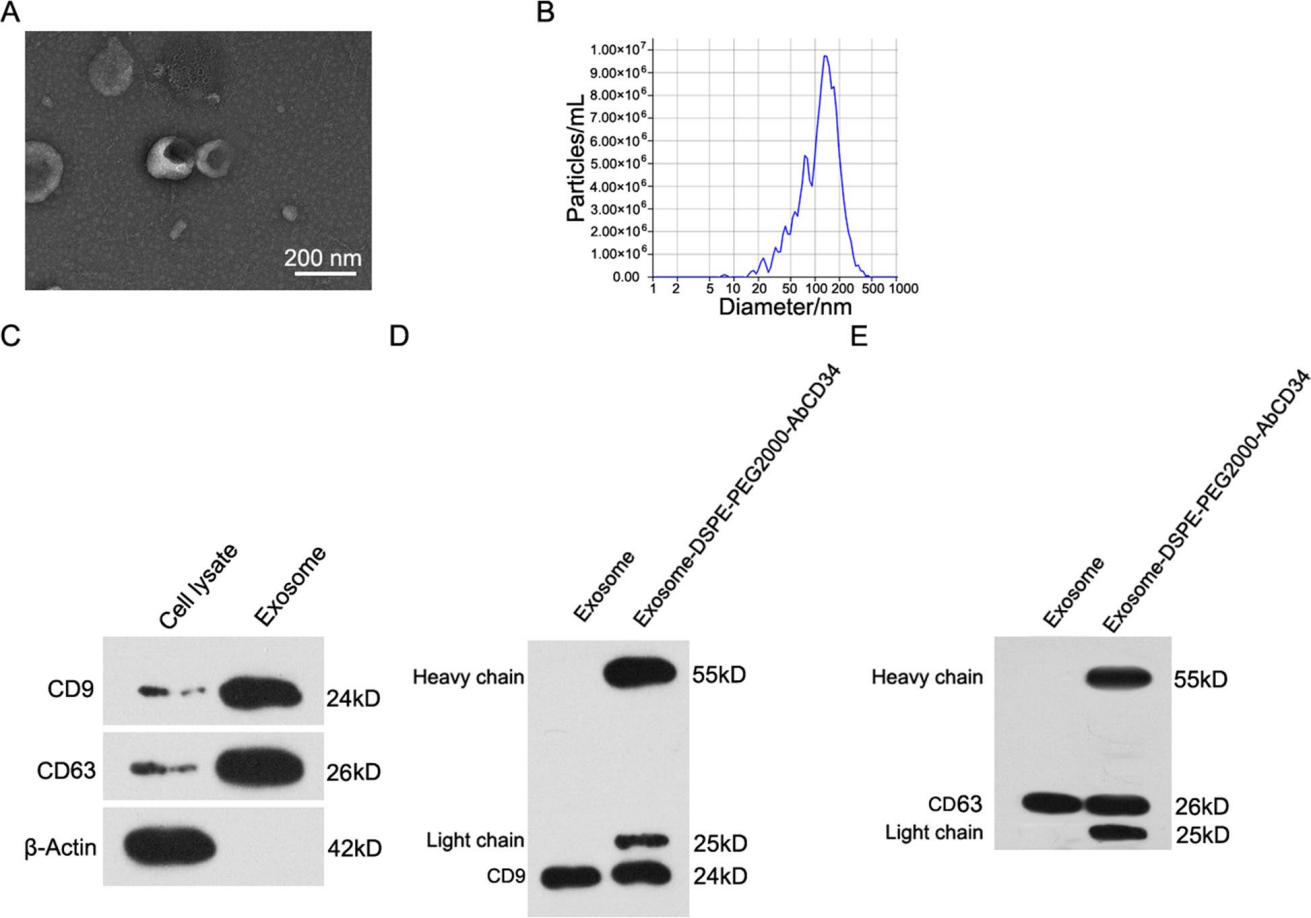
Preparation of and characterization of Exosome-DSPE-PEG-AbCD34. **A** The TEM image of exosome isolated from MAEC cells (**B**) The size of exosome tested by potential particle size analyzer (**C**) Western blot assay was used to detect exosome marker proteins (CD9, CD63) (**D**) Western blot assay was used to detect CD34 antibody loaded on the outer membrane of the exosome and CD9 in the exosomes, (**E**) Western blot assay was used to detect CD34 antibody loaded on the outer membrane of the exosome and CD63 in the exosomes.

Exosomes can be used as natural endogenous material carriers, and exosomes loaded with drugs have the advantages of low toxicity, no immunogenicity and good permeability. In this study, we first isolated exosomes from the MAEC culture medium of mouse aortic endothelial cells, and then synthesized Exosome-DSPE-PEG-Ab_CD34_ by inserting the synthesized DSPE-PEG-Ab_CD34_ into the exosome membrane structure. The exosomes were characterized by TEM (Fig. 1A) and potential particle size analyzer (Fig. 1B), the average diameter of prepared exosomes was about 150 nm. Western blot assay was used to detect exosome marker proteins (CD9, CD36) (Fig. 1C), as showed in the result, with CD9 and CD36 can be tested in exosome, meaning that the exosome was successfully extracted. CD34 antibody, which loaded on the outer membrane of the exosome, was detected by western blot assay, too (Fig. 1D, E). And the heavy chain and light chain of antibody was only showed in the exosome-DSPE-PEG-AbCD34. These results showed that we had successfully isolated and extracted MAEC exosomes from mouse aortic endothelial cells, and synthesized Exosome-DSPE-PEG-AbCD34 with CD34 targeting property.

### Biocompatibility, stability, targeting property and cell internalization of Exosome-DSPE-PEG-Ab_CD34_

**Fig. 2:**
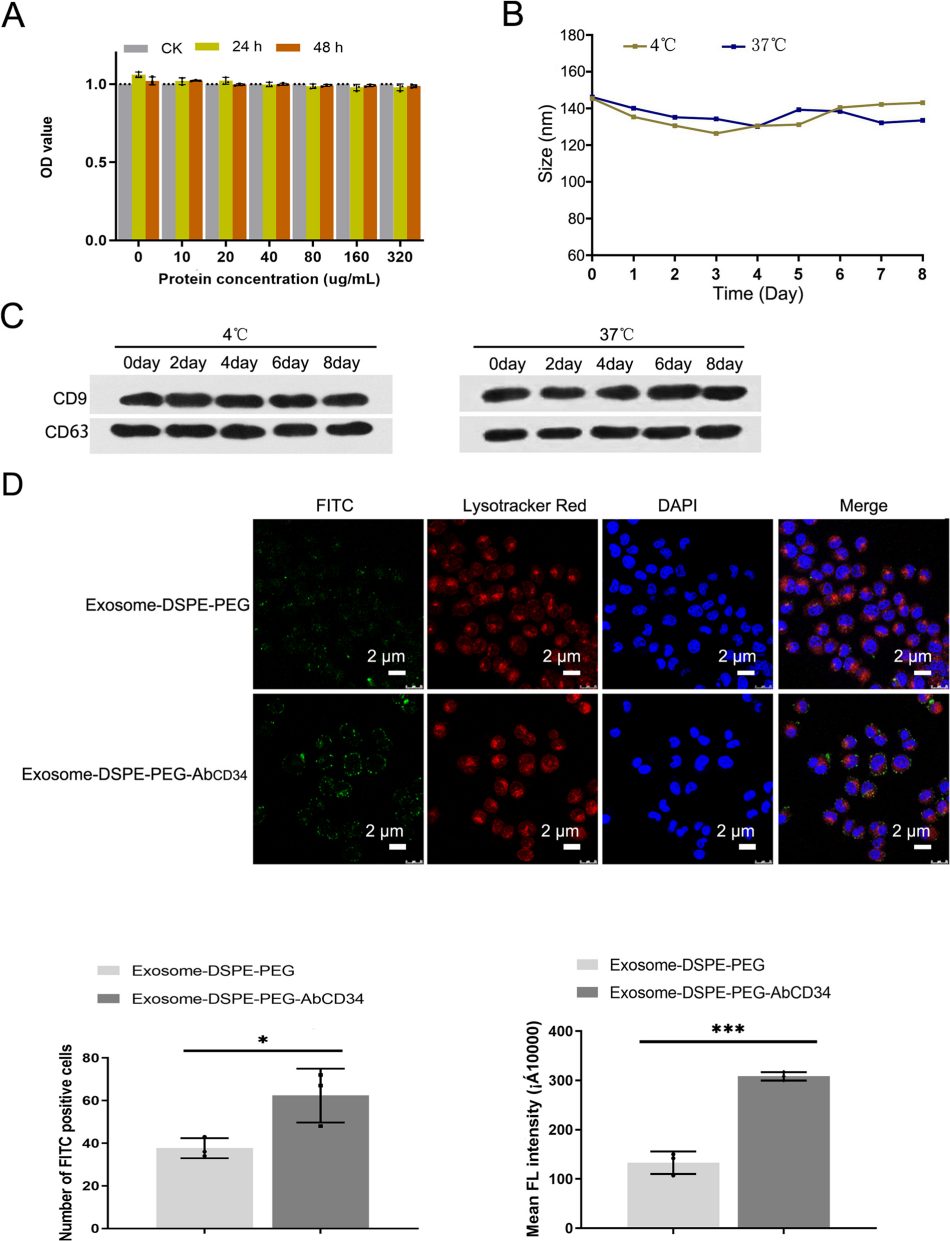
Biocompatibility, stability, targeting property and cell internalization of Exosome-DSPE-PEG-AbCD34. **A** Biocompatibility of Exosome-DSPE-PEG-AbCD34 to MAEC cells was tested by CCK-8 assay; **B** Stability of Exosome-DSPE-PEG-AbCD34 at 4 °C and 37 °C in different time; **C** western blot assay was used to detected the level of CD9 and CD36 at 4°C and 37°C in different time; **D** Confocal microscopy images of cell internalization testing of Exosome-DSPE-PEG-AbCD34 at ×20 magnification. The bar plot below shows the number of FITC positive cells and the calculated mean fluorescence of FITC intensity of each group. Scale bars: 2 μm.

Biocompatibility, stability, targeting and cell internalization are important characteristics of gene carriers. We used the CCK-8 assay to detect the effect of different concentrations of Exosome-DSPE-PEG-Ab_CD34_ on the viability of MAEC cells. The results showed that treatment with the concentration from 10 to 320 ug/ml of Exosome-DSPE-PEG-Ab_CD34_ had a negligible effect on cell proliferation and viability of MAEC cells (Fig. 2A). Then we tested the stability of Exosome-DSPE-PEG-Ab_CD34_ at 4°C and 37°C by DLS and western blot assay (Fig. 2B, C). Results showed that after being stored at 4°C and 37°C for 8 days, the particle size of exosome-DSPE-PEG-AbCD34 could stably maintained at around 140 nm, which means the Exosome-DSPE-PEG-AbCD34 can be stably stored at 4°C and 37°C for at least 8 days. Then we observed the targeting effect of exosome-DSPE-PEG-Ab_CD34_ on MAEC cells by laser confocal microscopy. The results showed that more FITC labeled exosome-DSPE-PEG-Ab_CD34_ could be observed on the surface and inside of MAEC cells after the exosome vector and cells were incubated for 48 hours, which indicates that exosome-DSPE-PEG-Ab_CD34_ has better cell targeting performance and cell internalization than exosome-DSPE-PEG (Figure 2D).

### Synthesis of Exosome-DSPE-PEG-Ab_CD34_-*Smad7* and its effect on SMAD7 expression in MAEC cells

**Fig. 3:**
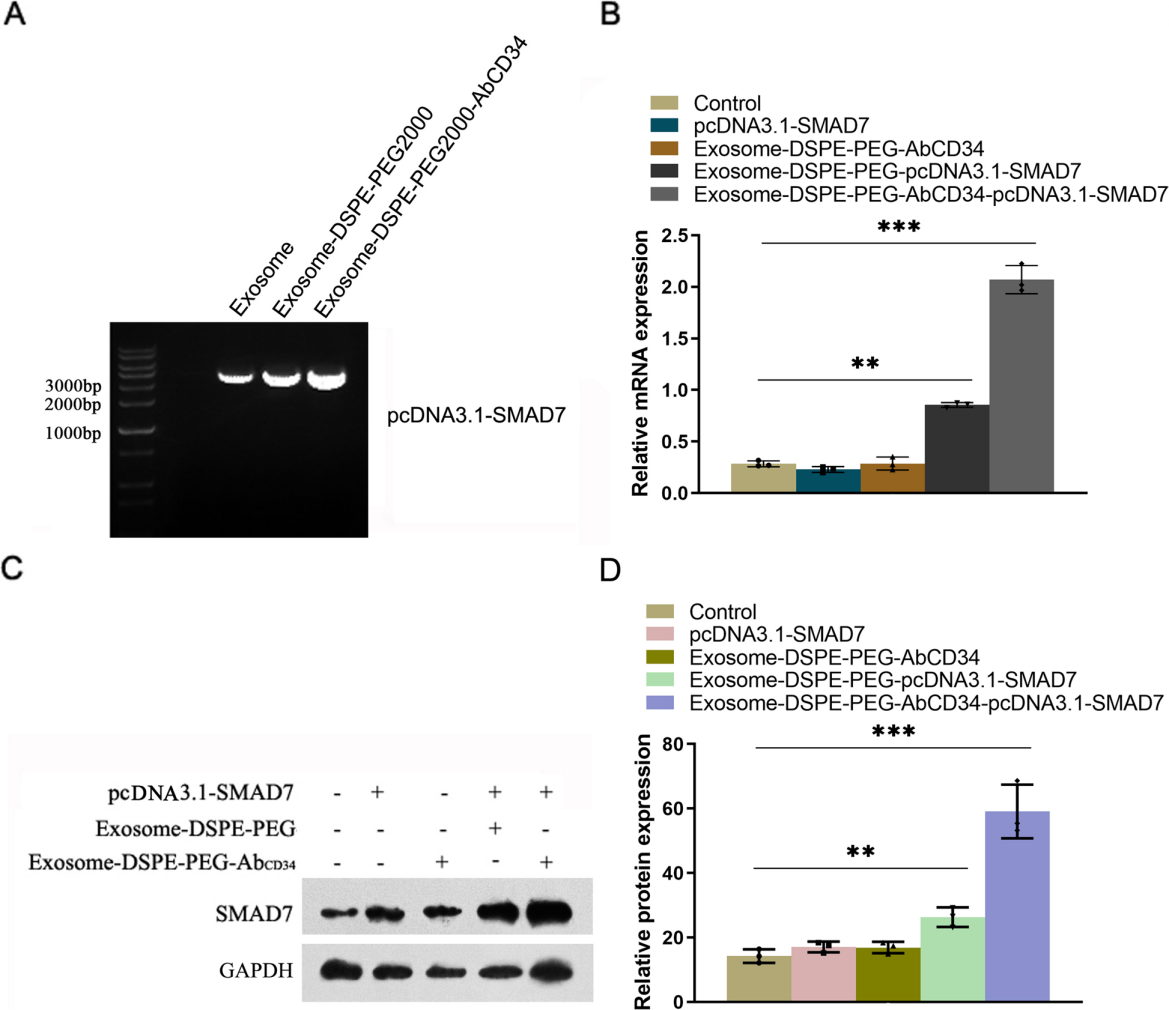
Synthesis of Exosome-DSPE-PEG-AbCD34-Smad7 and its effect on SMAD7 expression in MAEC cells. **A** DNA gel electrophoresis was used to detect the loading of pcDNA3.1-Smad7 in the exosome, Exosome-DSPE-PEG and Exosome-DSPE-PEG-AbCD34; **B** qPCR assay was used to test the gene expression level of Smad7 after exosome, Exosome-DSPE-PEG and Exosome-DSPE-PEG-AbCD34 was co-cultured with MAEC cells for 48 h (**C**, **D**) Western blot assay was used to test the protein expression of SMAD7 after exosome, Exosome-DSPE-PEG and Exosome-DSPE-PEG-AbCD34 was co-cultured with MAEC cells for 48 h.

We incubated the *Smad7* plasmid with Exosome-DSPE-PEG-AbCD34 to synthesize Exosome-DSPE-PEG-Ab_CD34_- *Smad7*, and then DNA gel electrophoresis was used to detect the loading of pcDNA3.1- *Smad7* in the Exosome-DSPE-PEG-Ab_CD34_- *Smad7* (Figure 3A). The result showed that pcDNA3.1- *Smad7* can be loaded in the exosome, Exosome-DSPE-PEG and Exosome-DSPE-PEG-AbCD34. After exosome-DSPE-PEG-Ab_CD34_- *Smad7* was co-cultured with MAEC cells for 48 h, the gene level of SMAD7 in MAEC cells was detected by qPCR and western blot. The results showed that Exosome-DSPE-PEG-Ab_CD34_ could transport pcDNA3.1- *Smad7* into MAEC cells and overexpress SMAD7 successfully (Figure 3B, C and D).

### The effect of Exosome-DSPE-PEG-Ab_CD34_-*Smad7* on EndMT pathway in MAEC cells

**Fig. 4:**
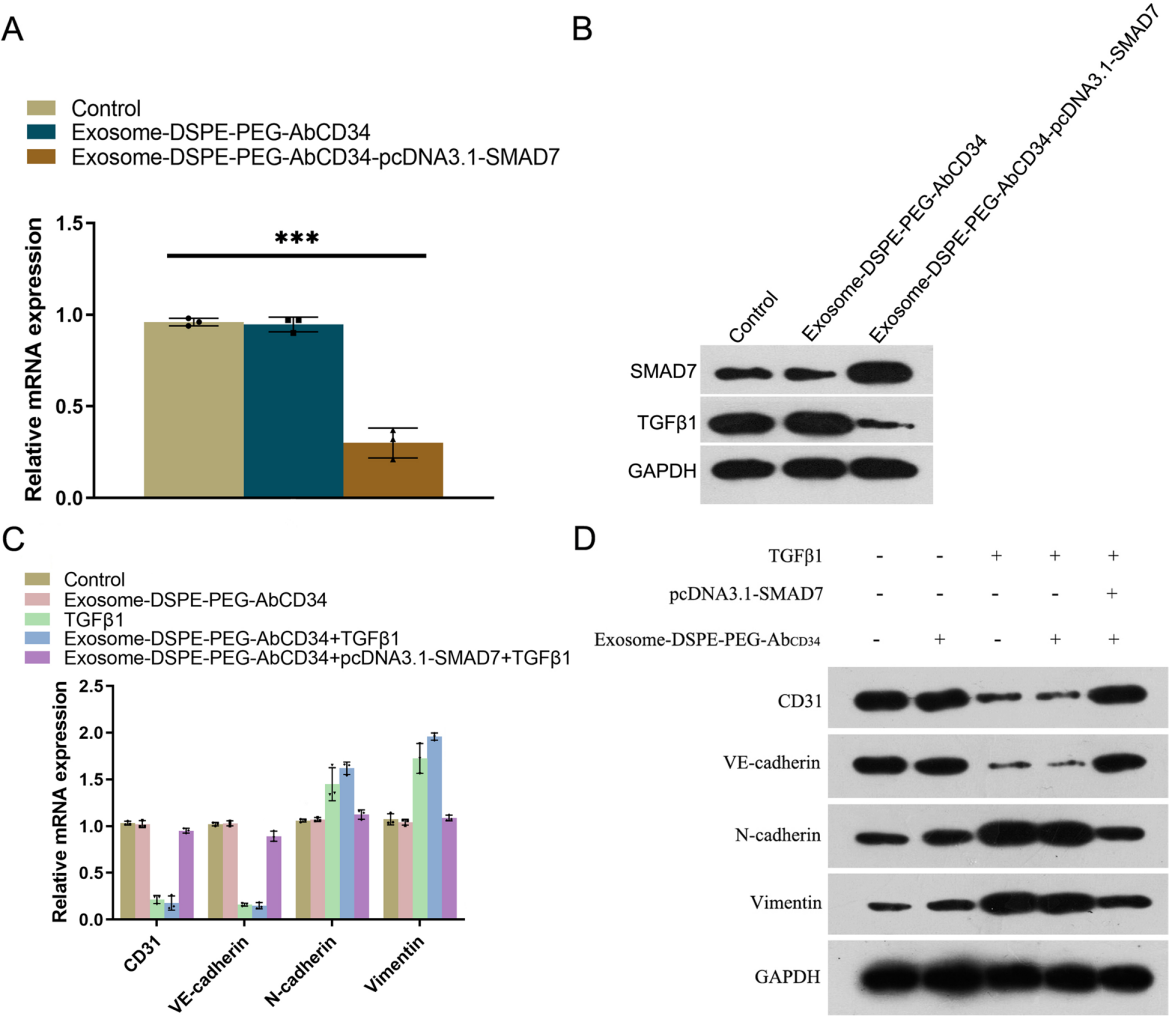
The effect of Exosome-DSPE-PEG-AbCD34-Smad7 on EndMT pathway in MAEC cells. **A** qPCR assay was used to test the effect of Exosome-DSPE-PEG-AbCD34-Smad7 on the expression of TGF-β1 (**B**) Western blot assay was used to test the effect of Exosome-DSPE-PEG-AbCD34-Smad7 on the expression of TGF-β1, qPCR and western blot assay was used to test the mRNA expression (**C**) and protein expression (**D**) of EndMT pathway related genes (CD31, VE-cadherin, N-cadherin and vimentin).

The EndMT has been proved to be an important mechanism in the pathogenesis of HO. In its molecular pathophysiological mechanism, the TGF-β (transforming growth factor-β) superfamily signaling pathway plays a leading role, and *Smad7* is an important suppressor gene in this signaling pathway. To determine the inhibiting effect of Exosome-DSPE-PEG-AbCD34-SMAD7 on the EndMT of MAECS, we stimulated MAECS with 10 ng/ml TGF-β1 for 48 h to simulate the effect of TGF-β1 on local EndMT at the cellular level with or without the administration of Exosome-DSPE-PEG-AbCD34- *Smad7*. The effect of Exosome-DSPE-PEG-Ab_CD34_-*Smad7* on MAECS was detected by qPCR and western blot. The results showed that TGF-β1 could downregulate CD31 and VE-cadherin, and up regulate N-cadherin and vimentin. Compared with the control group, the Exosome-DSPE-PEG-Ab_CD34_-*Smad7* transfection group could apparently decrease the level of TGF-β1 in MAECs. Furthermore, the Exosome-DSPE-PEG-AbCD34-*Smad7* can upregulate the expression of CD31 and VE-cadherin. Meanwhile, it also downregulated the expression of N-cadherin and vimentin (Figure 4C and D), which means it can then counteract the effect of TGF-β1 induced EndMT in MAECs. In conclusion, the overexpression of the SMAD7 induced by Exosome-DSPE-PEG-Ab_CD34_-*Smad7* can effectively reverse the EndMT pathway of MAECS mediated by TGF- β1.

## Discussion

Heterotopic ossification (HO) refers to the formation of atypical bone in muscle or at other sites of connective tissue, and HO which occurs around joints will develop clinical symptoms [25–27]. As a pathological bone formation, HO has an incidence rate vary from 9%-90%, and it can be influenced by several risk factors [4]. Severe HO can limit joint activity, even causing ankylosis and loss of joint function. It can also cause neurological symptoms and local pressure ulcers due to nerve wrapping [28]. Dyspareunia caused by heterotopic ossification of the adductor longus is also a rare complication [29]. Dynamic histomorphometry showed that compared with normal bone, ectopic ossified bone had higher metabolic activity, increased bone deposition rate, wider bone suture, and larger number of osteoblasts [30]. The fate of progenitor cells such as mesenchymal stem cells can be affected by stimulation from microenvironment [31, 32], and it has been proved that progenitor cells, inflammation niches and disorder of signaling pathway promote the formation of HO. Based on previous studies of HO related molecular mechanism and signaling pathway, we studied the targeted gene therapy method that may be used for the treatment of HO.

There were a plenty of studies indicating that the endothelial to mesenchymal transition (EndMT) plays an important role in the pathogenesis of HO. Under the regulation of inflammatory factors and the local neuroendocrine system, vascular endothelial cells pass through the vascular barrier, dissociate into tissues, and dedifferentiate into mesenchymal stem cells, then differentiate into chondrocytes and osteocytes under local conditions, and form HO tissue through the process of intramembrane osteogenesis and chondrogenic osteogenesis. EndMT was mainly induced by the TGF-β superfamily signaling pathway, and SMAD7 is an important suppressor gene of the TGF-β signaling pathway. Previous studies have shown that SMAD7 has the potential to prevent myofibroblast transformation and other EndMT progression, which verifies that high expression of SMAD7 in endothelial cells can effectively reverse the TGF-β1 mediated EndMT pathway of RAOECS. This result indicates that SMAD7 can be used in the prevention and treatment of HO [7]. The *Smad7* gene needs the help of a gene vector with high biocompatibility and good targeting property to enter into the cells. Exosomes are small vesicles with a diameter of 30-150 nm naturally secreted by cells [33], and they can be used as potential carriers to deliver drugs, therapeutic genes or proteins in clinical treatment due to their stability and high biocompatibility [34–37]. In current study, the exosomes produced from mouse aortic endothelial cells were selected as vehicles of gene therapy as it mirror the marker molecules of parent cells and was associated with many physiological progresses such as anti-inflammation, anti-oxidation and the inhibition of endothelial-to-mesenchymal transition (EndMT) [12, 22]. However, normal exosomes cannot target specific cells. Loading targeted peptides with amphiphilic groups on the surface of exosomes can improve the targeting of exosomes.

The hydrophilic peptides always need to be modified by liposoluble compounds to integrate into the hydrophobic membrane of exosomes, which are mainly composed of lipid membrane. In this study, we used DSPE-PEG2000-NHS to bind CD34 antibody (AbCD34) to the surface of secreted exosomes to synthesize an exosome vector with a high endothelial cell targeting property (Exosome-DSPE-PEG-AbCD34). The assays we performed to detect its physical and biological characteristics showed that Exosome-DSPE-PEG-AbCD34 had no effect on MAEC cell viability at a high concentration, and Exosome-DSPE-PEG-AbCD34 could be stably stored at 4°C as well as room temperature for at least 8 days. Moreover, compared with Exosome-DSPE-PEG, Exosome-DSPE-PEG-AbCD34 has a better targeting property to MAEC cells and can enter into endothelial cells more effectively, which confirmed that the modification with antibody CD34 can remarkably develop exosomes’ ability to internalize into endothelial cells. Then we loaded *Smad7* plasmid into the Exosome-DSPE-PEG-AbCD34 to compose the Exosome-DSPE-PEG-Ab_CD34_- *Smad7*, and the transfection assay showed that Exosome-DSPE-PEG-Ab_CD34_-*Smad7* could significantly increase the level of SMAD7 in MAEC cells.

Finally, we examined the effect of Exosome-DSPE-PEG-Ab_CD34_-*Smad7* on the molecules related to the EndMT pathway in MAEC cells. The results showed that the Exosome-DSPE-PEG-Ab_CD34_-*Smad7* transfection group can up regulate the expression of CD31 and VE-cadherin, which proved that the Exosome-DSPE-PEG-AbCD34-*Smad7* can counteract the effect of TGF-β1. Meanwhile, Exosome-DSPE-PEG-Ab_CD34_-*Smad7* transfection also downregulated the expression of N-cadherin and vimentin.

## Conclusions

In conclusion, the overexpression of SMAD7 induced by Exosome-DSPE-PEG-Ab_CD34_-*Smad7* can effectively reverse the EndMT pathway of MAEC mediated by TGF- β1. These results indicate that Exosome-DSPE-PEG-AbCD34-*Smad7* may has the potential for the prevention and treatment of HO. This study provides a theoretical basis and gene therapy strategy for the prevention and treatment of HO. Whether this modified method may be suitable for other sort of exosome and the concrete effect of Exosome-DSPE-PEG-AbCD34-*Smad7* on prevention of HO progression should be studied in further research.

## Supplementary Information

**Additional file 1.**

## Data Availability

The datasets generated and/or analyzed during the current study are available from the corresponding author by reasonable request.

## Abbreviations

HO: Heterotopic ossification
EndMT: Endothelial to mesenchymal transition
SMAD7: Mothers Against Decapentaplegic Homolog 7
MAECs: Mouse aortic endothelial cells
TEM: Transmission electron microscopy
