# Supplementary figures and images for: Exosomes for gene therapy effectively inhibit the endothelial-mesenchymal transition in mouse aortic endothelial cells
Source: BMC Musculoskelet Disord. 2021 Nov 30;22:1000. doi: 10.1186/s12891-021-04896-0 (PMC8630863; doi:10.1186/s12891-021-04896-0)

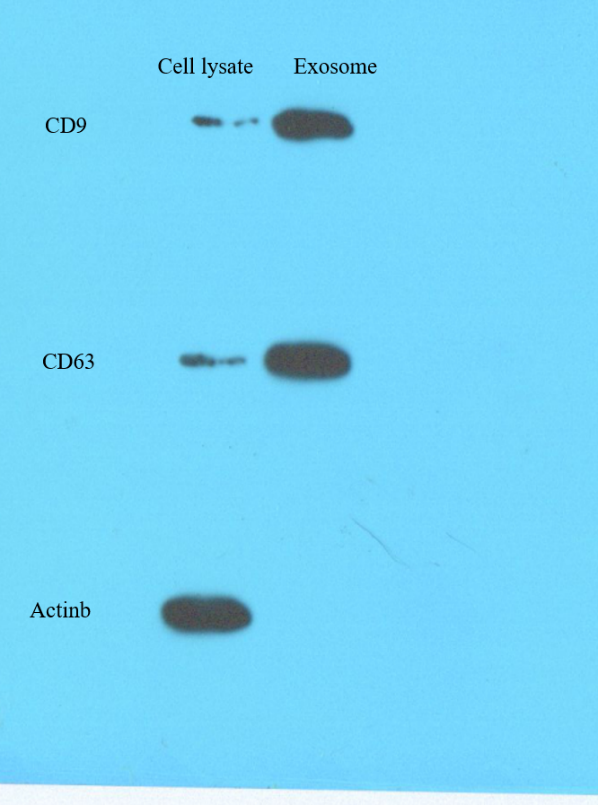


Figure 1C


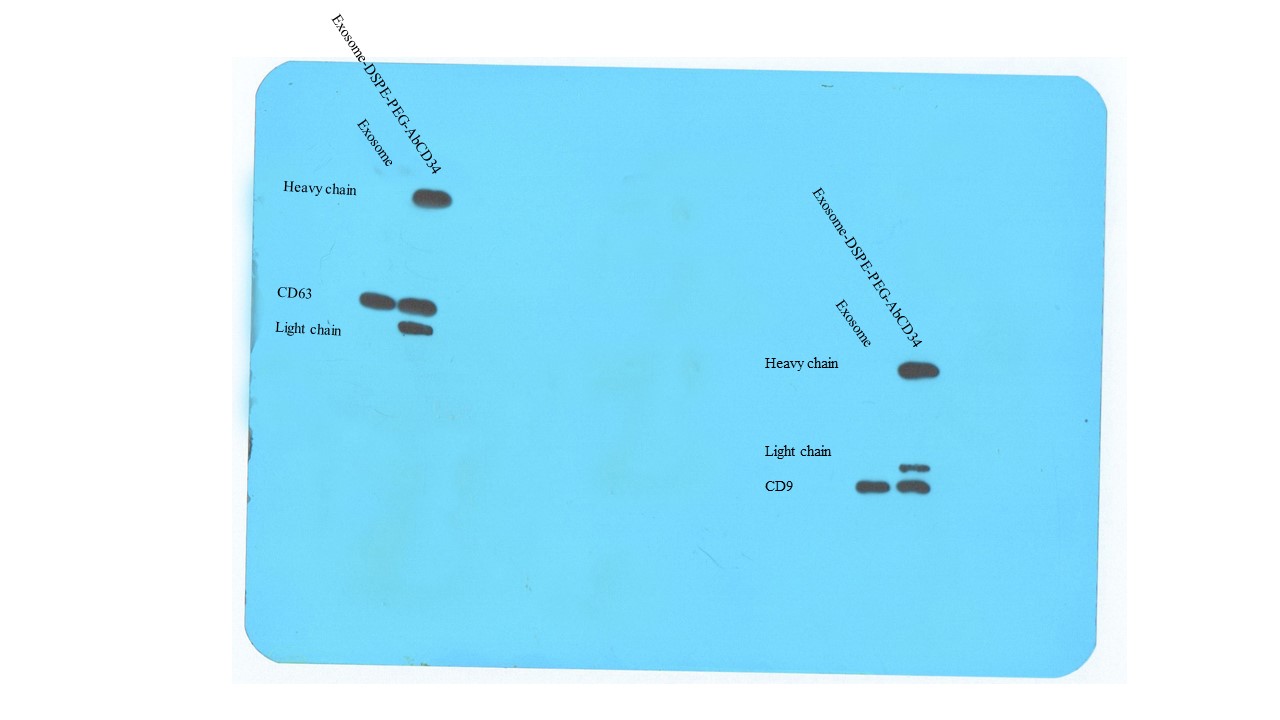


Figure 1E，Figure 1D


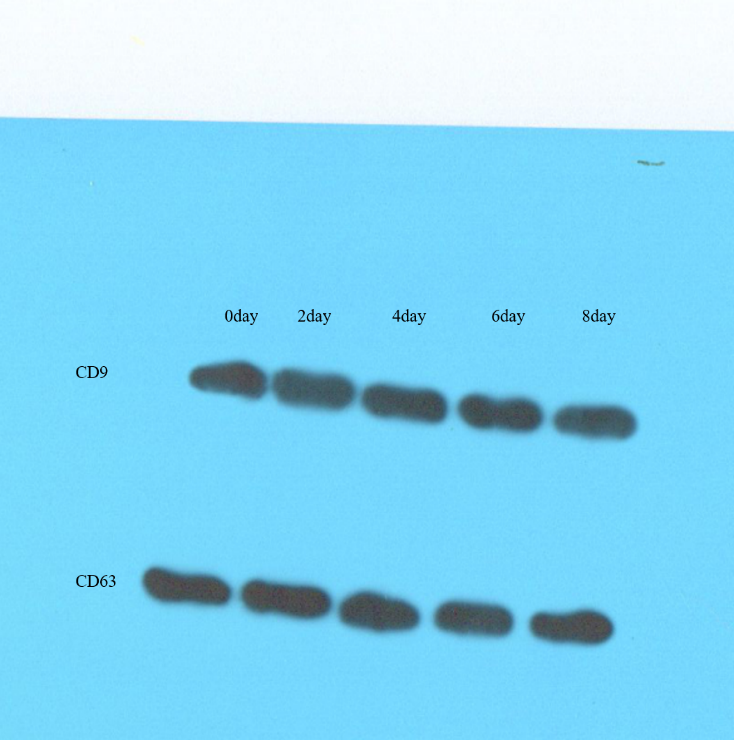


Figure 2C-1


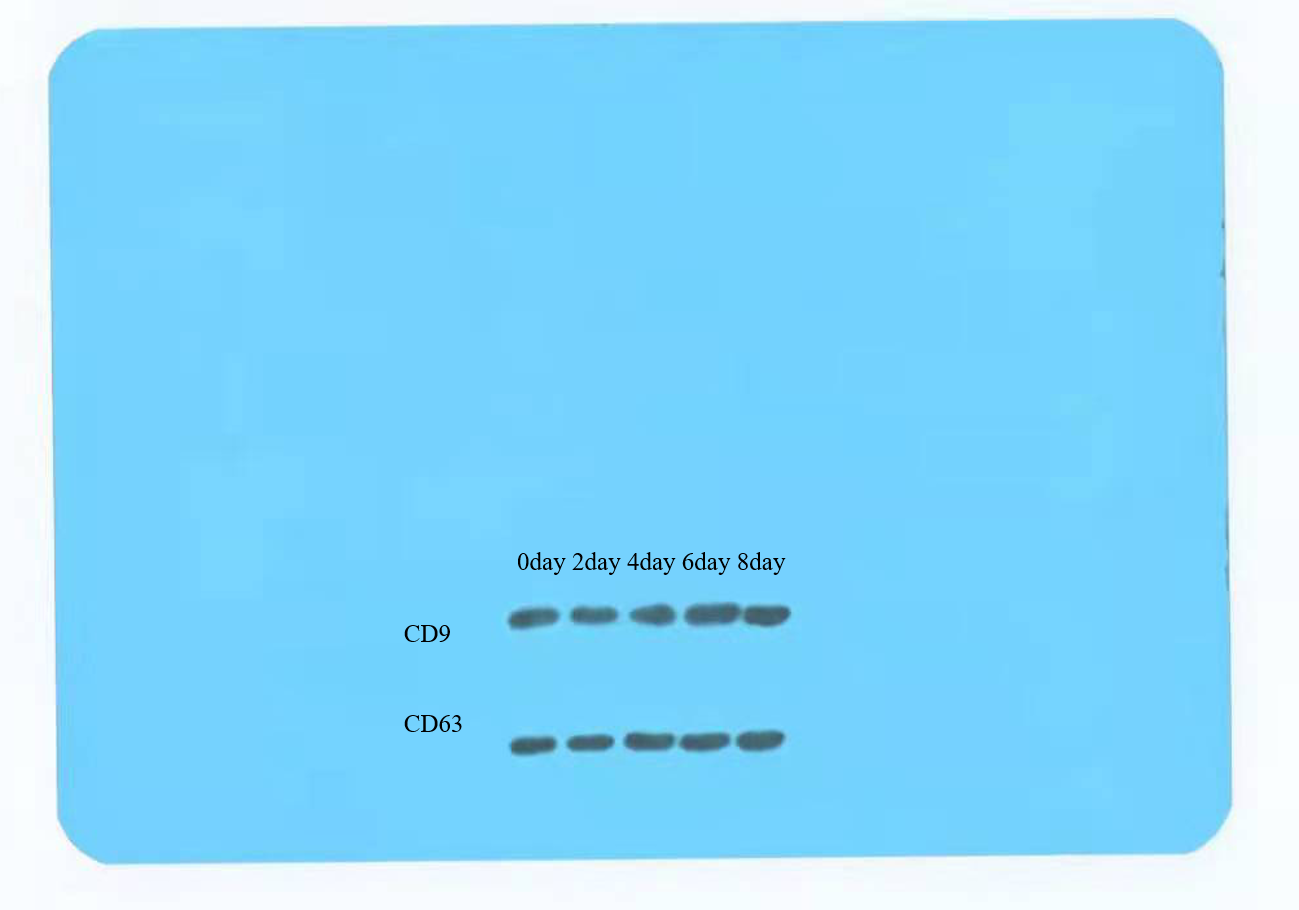


Figure 2C-2


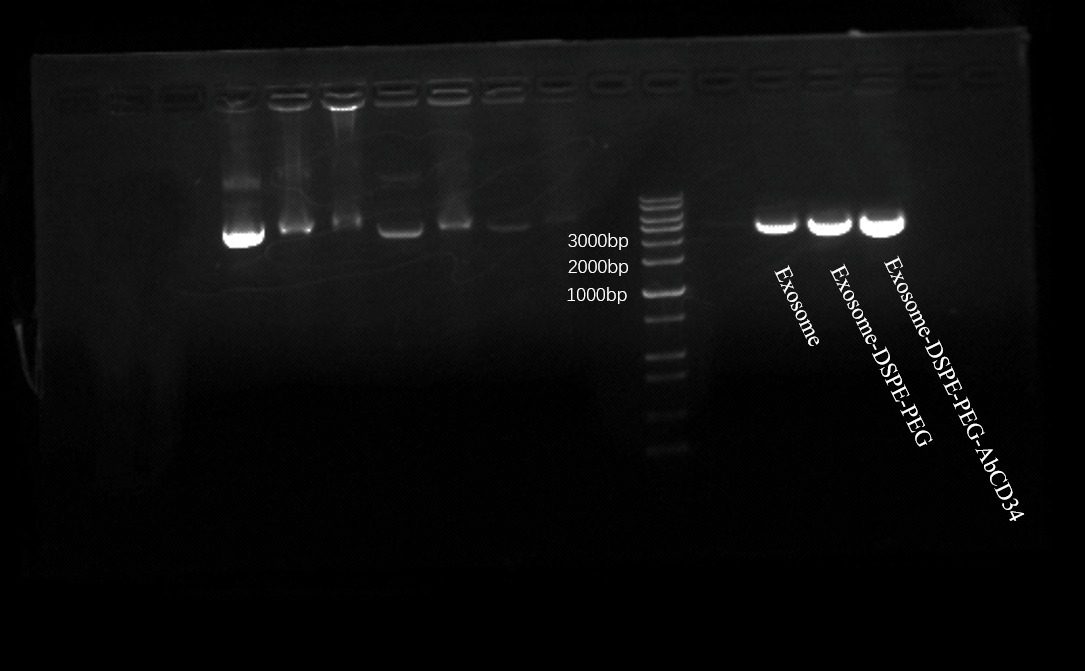


Figure 3A


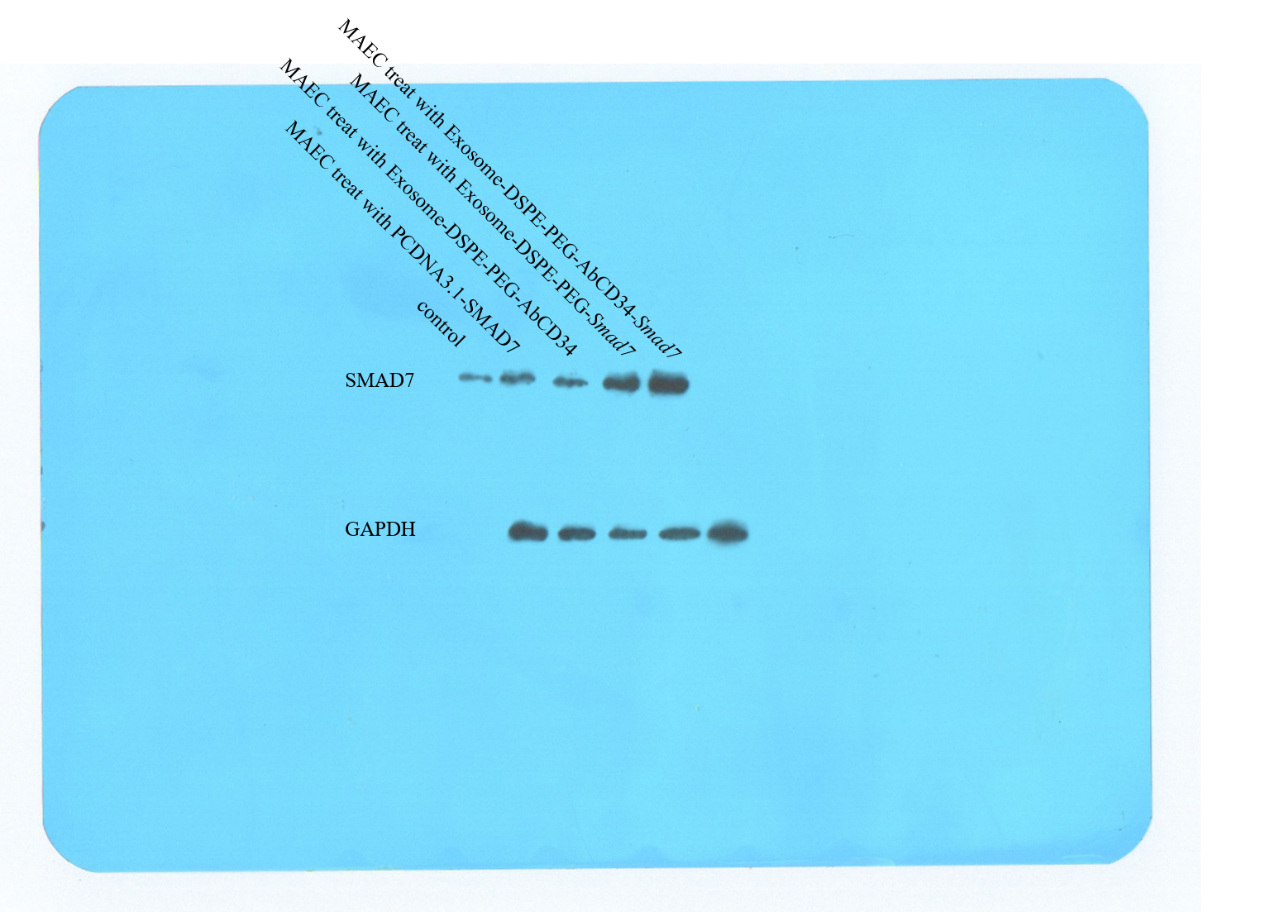


Figure 3C


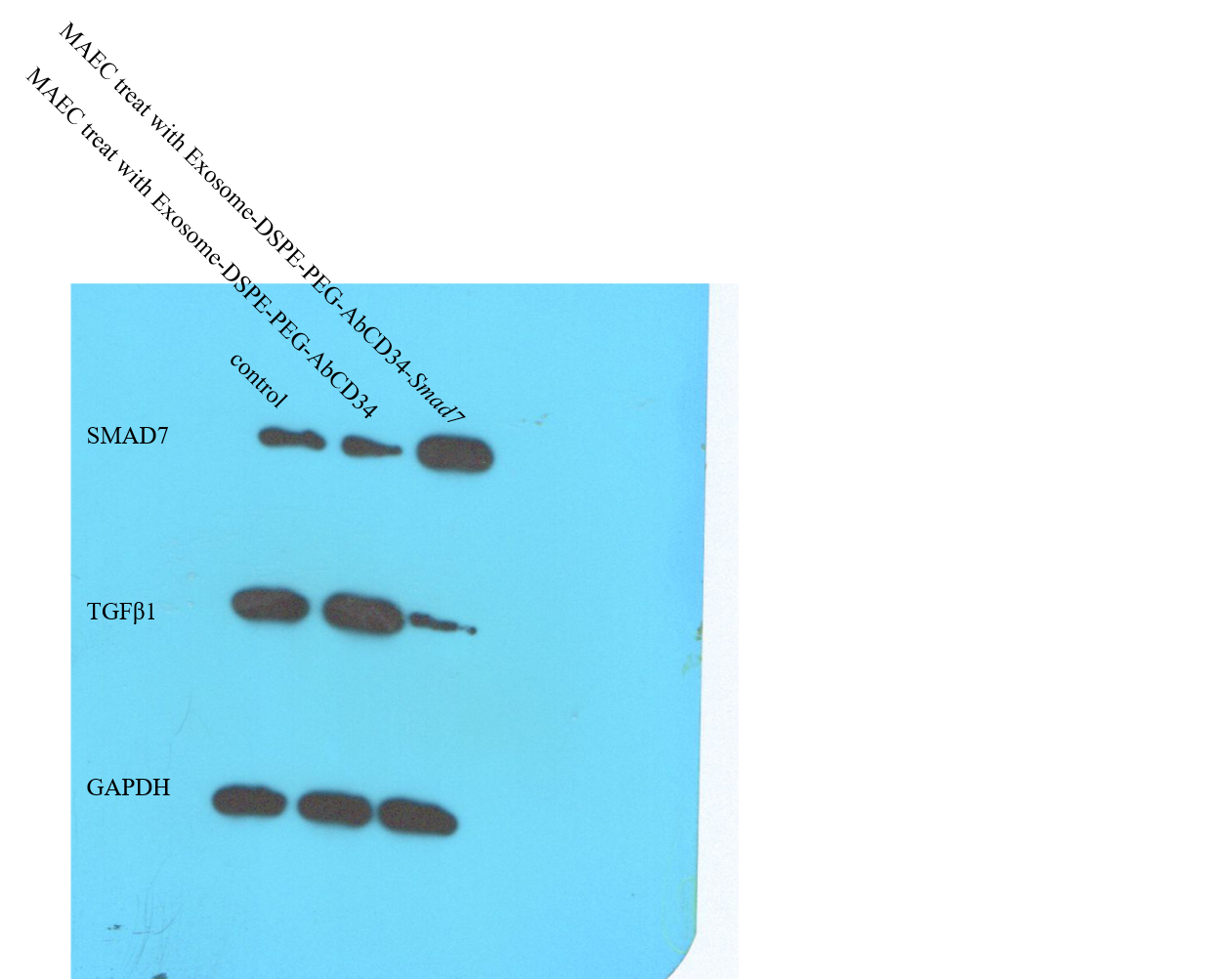


Figure 4B


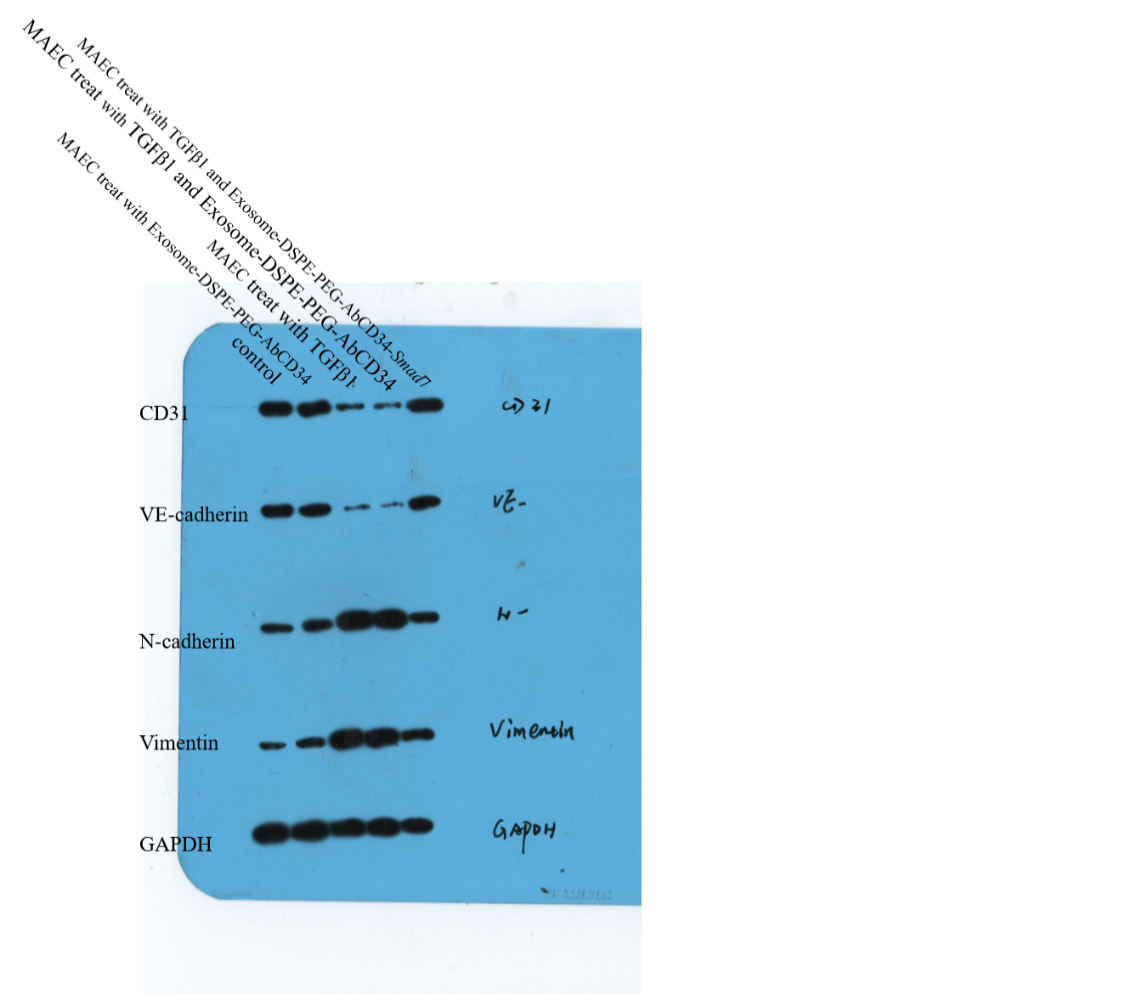


Figure 4D

Supplement: Supplementary file 1 — Additional file 1. [file 12891_2021_4896_MOESM1_ESM.docx]
